# Toll-Like Receptor Agonists as Adjuvants for Allergen Immunotherapy

**DOI:** 10.3389/fimmu.2020.599083

**Published:** 2020-11-12

**Authors:** Max E. Kirtland, Daphne C. Tsitoura, Stephen R. Durham, Mohamed H. Shamji

**Affiliations:** ^1^ Immunomodulation and Tolerance Group, Allergy and Clinical Immunology, Inflammation, Repair and Development, National Heart and Lung Institute, Imperial College London, London, United Kingdom; ^2^ NIHR Biomedical Research Centre, Asthma UK Centre in Allergic Mechanisms of Asthma Imperial College London, London, United Kingdom; ^3^ Inflammation, Repair and Development, National Heart and Lung Institute, Imperial College London, London, United Kingdom

**Keywords:** adjuvants, allergen-specific immunotherapy, toll-like receptors, immunomodulation, allergic rhinitis****

## Abstract

Toll-like receptors (TLRs) are essential components of innate immunity and provide defensive inflammatory responses to invading pathogens. Located within the plasma membranes of cells and also intracellular endosomes, TLRs can detect a range of pathogen associated molecular patterns from bacteria, viruses and fungi. TLR activation on dendritic cells can propagate to an adaptive immune response, making them attractive targets for the development of both prophylactic and therapeutic vaccines. In contrast to conventional adjuvants such as aluminium salts, TLR agonists have a clear immunomodulatory profile that favours anti-allergic T lymphocyte responses. Consequently, the potential use of TLRs as adjuvants in Allergen Immunotherapy (AIT) for allergic rhinitis and asthma remains of great interest. Allergic Rhinitis is a Th2-driven, IgE-mediated disease that occurs in atopic individuals in response to exposure to otherwise harmless aeroallergens such as pollens, house dust mite and animal dander. AIT is indicated in subjects with allergic rhinitis whose symptoms are inadequately controlled by antihistamines and nasal corticosteroids. Unlike anti-allergic drugs, AIT is disease-modifying and may induce long-term disease remission through mechanisms involving upregulation of IgG and IgG4 antibodies, induction of regulatory T and B cells, and immune deviation in favour of Th1 responses that are maintained after treatment discontinuation. This process takes up to three years however, highlighting an unmet need for a more efficacious therapy with faster onset. Agonists targeting different TLRs to treat allergy are at different stages of development. Synthetic TLR4, and TLR9 agonists have progressed to clinical trials, while TLR2, TLR5 and TLR7 agonists been shown to have potent anti-allergic effects in human *in vitro* experiments and *in vivo* in animal studies. The anti-allergic properties of TLRs are broadly characterised by a combination of enhanced Th1 deviation, regulatory responses, and induction of blocking antibodies. While promising, a durable effect in larger clinical trials is yet to be observed and further long-term studies and comparative trials with conventional AIT are required before TLR adjuvants can be considered for inclusion in AIT. Here we critically evaluate experimental and clinical studies investigating TLRs and discuss their potential role in the future of AIT.

## Introduction

Allergic rhinitis (AR) affects an estimated 400 million people worldwide and up to 26% of adults within the UK (1, 2). While symptoms such as rhinorrhoea and itching are not life-threatening, they have significant socioeconomic consequences through reduced productivity at work and school, as well as impacts on quality of life through loss of sleep (3). The prevalence of AR is increasing globally (2), with this trend being linked with factors such as increased socioeconomic status (4), a reduction in chronic infectious diseases, and the adoption of a “westernised” lifestyle (5). The use of symptomatic pharmacotherapy such as antihistamines and nasal corticosteroids to relieve AR is common and associated with considerable relief in the majority of cases other than the most severe. However, sustained clinical benefit requires the continuous use of medication and is not associated with alterations in the causal mechanisms of the disease. As avoidance is not always a viable option in AR, Allergen Immunotherapy (AIT) is effective when symptomatic treatments are inadequate to control symptoms (6–8). AIT is the only disease-modifying treatment available for AR, and current delivery methods of AIT are either the subcutaneous or sublingual routes. Subcutaneous immunotherapy requires monitoring in a clinical environment to identify early and treat occasional systemic reactions, whereas sublingual immunotherapy is suitable for home administration. Local reactions to both subcutaneous immunotherapy and sublingual immunotherapy are common, with occurrences documented in 82 and 75% patients respectively, and 3–4 fatalities per year worldwide being reported for subcutaneous immunotherapy (9). Both modalities of treatment when given over 3 years are disease-modifying and result in long-term benefits for years following their discontinuation. A recent study that assessed the impact of a shorter two-year course of treatment was highly effective but did not result in long-term tolerance (10). While immunotherapy is assuredly beneficial, and the only disease-modifying treatment currently available, improvements in safety and a shortened course of therapy would see an increased rate of patient acceptance. In this review, we report on how Toll-like receptor (TLR) agonists might provide a solution to this unmet need through co-administration or conjugation with an allergen. Understanding how TLRs modulate allergic inflammation while promoting immune tolerance will provide insights not only for allergic rhinitis and AIT but also for other allergic airway diseases.

## Overview of Allergen Immunotherapy and Toll-Like Receptors

### Tolerance Mechanisms in AIT

Immune tolerance induction during AIT is mediated through a variety of complex molecular, cellular, and humoral mechanisms that inhibit the onset of the early and late phase inflammatory response upon allergen exposure. A key factor in mediating mast-cell induced histamine release and clinical biomarkers of atopy are circulating IgE levels (11). IgE antibodies are produced by B cells that have undergone class-switching from IgM/IgG/IgA (12) following allergen-exposure, and stimulation by Th2 and T follicular helper cells (13). T follicular helper cells are located within the germinal centres of lymph nodes where they secrete IL-21 (14) to promote B cell differentiation into immunoglobulin secreting cells, with IL-4 specifically driving IgE production from B cells (15). IgE binds to its high-affinity FcϵRI on mast cells, sensitising them to the allergen. During the onset of tolerance in a clinical setting, there is an induction of allergen neutralising IgG and IgG4, antibodies with the capacity to prevent basophil activation and CD23-mediated IgE-facilitated allergen presentation that has been reported in several studies (16, 17).

**Figure 1:**
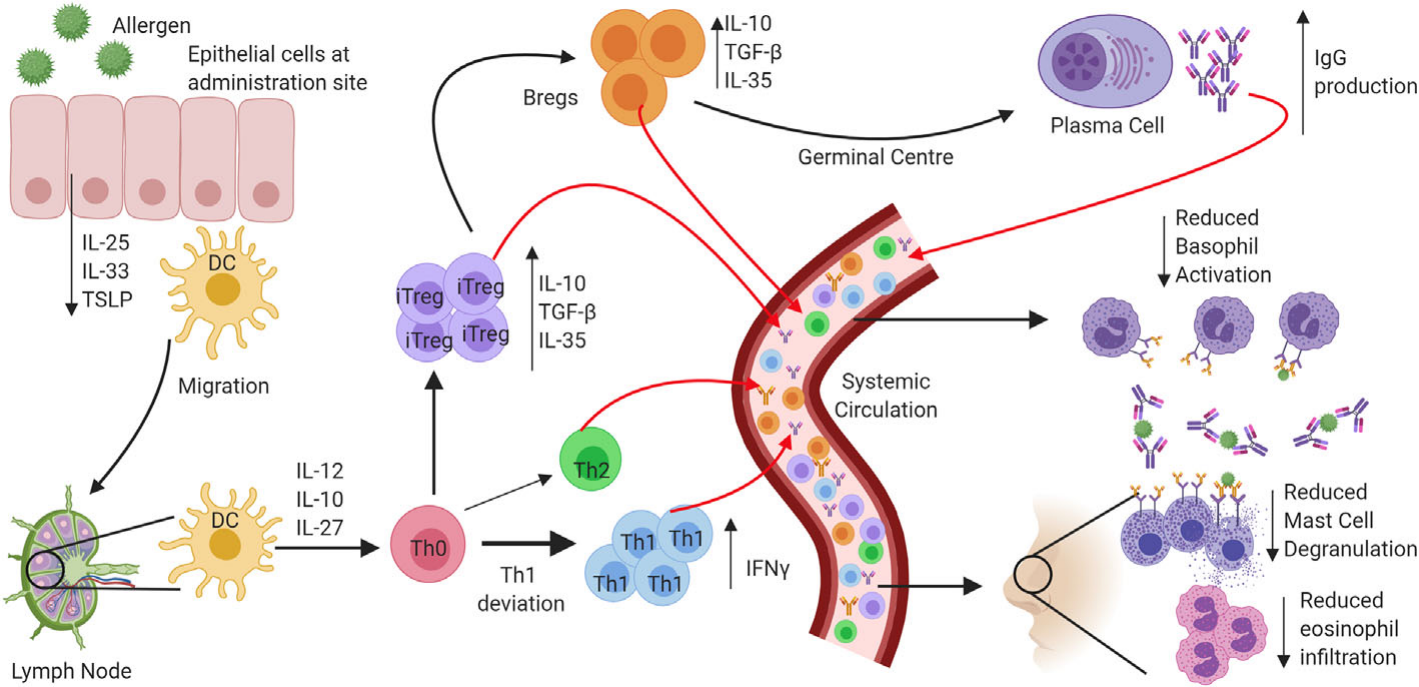
Mechanisms of tolerance onset following exposure to allergen through allergen immunotherapy. In atopic allergic individuals, following exposure to inhaled allergens the airway epithelium responds by the release of a panel of cytokines that include interleukin 25 (IL-25), Interleukin 33 (IL-33) and Thymic stromal lymphopoietin (TSLP). The resulting activation and maturation of dendritic cells following allergen stimulation results in preferential development of a pro-Th2 type T lymphocyte development. In contrast, following allergen immunotherapy, activated dendritic cells migrate to regional lymph nodes, and preferentially produce regulatory and Th1 skewing cytokines such as IL-10, IL-12, and IL-27. Priming of Naïve CD4^+^ helper cells promotes IFN-γ producing Th1 cells and IL-10, TGFβ, and IL-35 producing T regulatory cells. The induction of B regulatory cells also leads to IL-10 production and promotes the production of “blocking” IgG antibodies from B cells and that compete with IgE for allergen. The resulting prevention of IgE cross-linking on mast cells and basophils inhibits their activation and degranulation. Reduced production of Th2 cytokines results in inhibition of eosinophil activation and survival in the airways.

In addition to antibody-mediated effects, a key outcome in determining the onset of AIT-induced immune tolerance is a reduction in the number of circulating allergen-specific Th2 cells and the production of cytokines into the local milieu such as IL-4, IL-5, and IL-13 following allergen exposure. During subcutaneous immunotherapy and sublingual immunotherapy, both the number of allergen-specific Th2 cells and the local production of Th2-associated cytokines are reduced. Importantly, symptom relapse after discontinuation of AIT has been associated with a return to normal cytokine responses and numbers of peripheral Th2 cells. Eosinophilic infiltration during the late phase of the allergic response post allergen exposure is mediated through IL-5 secretion from Th2 cells, and this is reduced following AIT (18, 19). Numerous studies have also identified that local increases in regulatory T cells (Tregs) (20) secreting cytokines such as IL-10 (21), TGF-β (22), and IL-35 (23), are essential in promoting tolerance. This adaptive regulatory response is also observed in B cells, where both IL-10 secretion and IgG4 production from B regulatory cells, are crucial factors in the maintenance of tolerance (24, 25). Finally, the skewing of the immune response towards being Th1-mediated, something which can be induced by TLR activation, is a crucial factor in inducing and maintaining tolerance in AIT (21, 26, 27). A graphical summary of tolerance induction to allergen following AIT is in **Figure 1**. By utilising TLRs in AIT, this rate of Th1 skewing may be enhanced for faster tolerance onset.

### An Overview of TLRs

**Figure 2:**
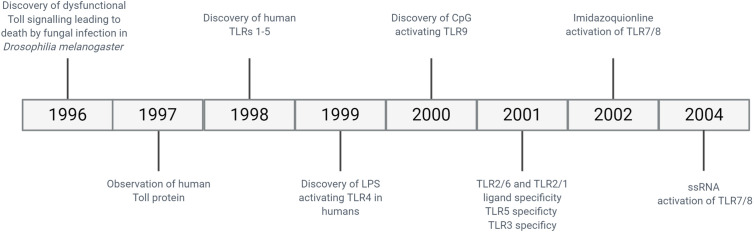
Timeline of discovery of TLRs and their ligands.

**Figure 3:**
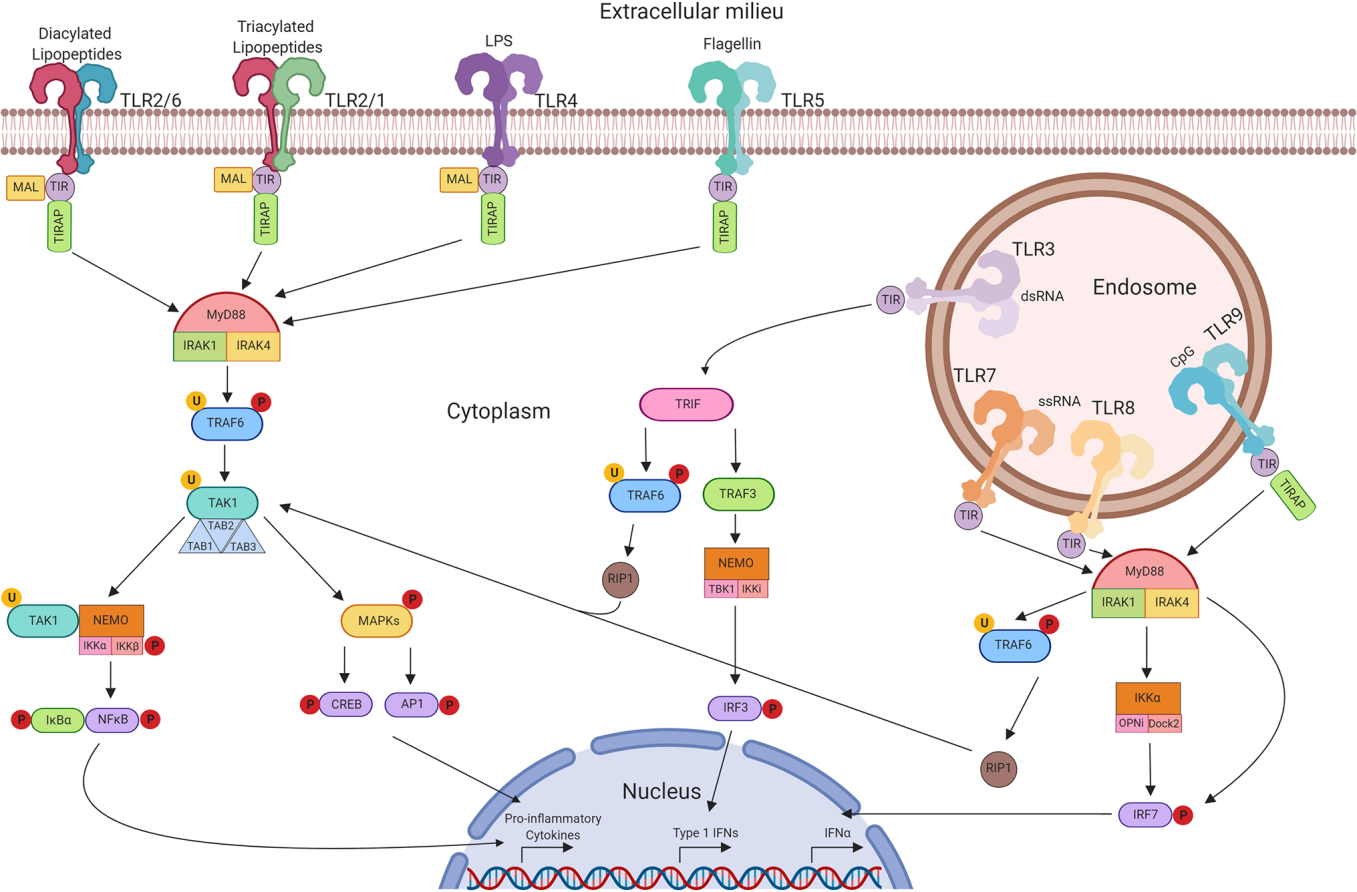
TLR signalling pathways. Extracellular TLRs 2/6, 2/1, 4, and 5 recognise bacterial diacylated lipopeptides, triacylated lipopeptides, LPS, and flagellin respectively. This activates downstream signalling through TIR leading to the formation of the MyD88 complex. This in turn activates TRAF6 and TAK1 and the formation of the TAB complex. TAK1 can then promote MAPK phosphorylation leading to the activation of CREB and AP-1 transcription factors. Alternatively, activation of NEMO leads to NFκB translocation to the nucleus. Unlike other TLRs, TLR3 signals through TRIF and can lead to the activation of TRAF6 and RIP-1, promoting CREB, AP-1, and NFκB through TAK1. Alternatively, activation of TRAF3 can signal through NEMO to promote translocation of IRF3. Intracellular TLRs 7, 8, and 9 signal through MyD88 upon activation to activate IKKα and IRF7—leading to the production of Type 1 IFNs.

The need to protect a host organism against a range of commonly encountered pathogens is an essential part of a functioning immune system, and characteristics of innate immunity are evolutionarily conserved between invertebrates and vertebrates. The functional importance of one component of the immune system, the Toll protein, was demonstrated in adult Drosophila melanogaster in which dysfunctional signalling following activation of Toll lead to premature death induced by fungal infection (28). It was then observed in humans that this series of proteins were highly conserved, with the identification of multiple TLRs containing similar sequence homology to Toll observed in Drosophila (29). The discovery of hToll was reported in 1997 (30), followed by TLRs 1–5 in 1998 (including hToll renamed to TLR4) (29), although their function was then unknown. Elucidation of TLR sensing in humans began with the discovery of lipopolysaccharide (LPS) mediating TLR4 activation in 1999 (31), with subsequent discoveries identifying the remaining TLRs and their ligands. This concluded with the identification of TLR7 and TLR8 sensing of single-stranded viral RNA in 2004 (32). The timeline of the discovery of TLRs and their ligands is summarised in **Figure 2**. TLRsare expressed on immune cells, including professional antigen-presenting cells such as dendritic cells (DCs), monocytes/macrophages, but also on T- and B-lymphocytes, as well as on non-immune cells such as epithelial cells, endothelial cells and fibroblasts (33). TLRs can be expressed on the plasma membrane of cells to detect extracellular pathogens or are located within the intracellular compartment, localised to the membranes of the endoplasmic reticulum, endosomes, and lysosomes. TLRs form dimers, of which TLR2 forms heterodimers with TLR1 or TLR6 while the other TLRs commonly form homodimers. All TLRs with the exception of TLR3 utilise the adaptor protein MyD88 to promote downstream signal propagation, whereas TLR3 exclusively utilises the TRIF protein. These responses are specialised further downstream to promote either inflammatory cytokine production or interferon production depending on the stimulus and the TLR propagating the signal. The signalling cascade following TLR activation is summarised in **Figure 3** (33–41). This induction of an initial innate immune response can also have more prolonged effects. Following activation by TLRs, dendritic cells (DCs) can act as a bridge between innate and adaptive immunity. This is a key advantage of utilising TLRs over conventional adjuvants alone, as the capacity to induce specific downstream T cell responses (42) can have applications in AIT to re-programme the immune response to allergen to a favourable Th1 response.

### Adjuvants Utilised in AIT

An ideal adjuvant, when combined with an allergy vaccine, should activate the innate immune system to amplify the downstream adaptive response to the antigen. The promotion of specific immunological memory and consequently, long-term immunity to the allergen is what makes AIT the only disease-modifying therapy. The most common adjuvants licensed for use in AIT in Europe and the US are aluminium salts (alum) or squalene-in-water emulsions (42). However, trends amongst immunotherapy regimens suggest that immunomodulators such as TLR agonists, and alternative adjuvants such as microcrystalline tyrosine (MCT) are becoming more prevalent. Allergen peptides are also becoming an area of increasing interest after demonstrating reduced allergenicity responses and upregulating Treg and B regulatory cell populations (43). It remains to be seen if peptide immunotherapy could benefit from the addition of an immunomodulatory agent such as a TLR agonist.

Alum is one of the longest used and most prevalent forms of adjuvant in modern vaccines and is currently used in a high proportion of subcutaneous immunotherapy formulations within Europe (44). An *in vitro* model of grass pollen allergy using PBMCs from grass pollen sensitive AR patients, showed that Alum decreased allergen-induced IL-5 and IL-13 and upregulated co-stimulatory markers (45). Alum demonstrates an enhanced safety profile over non-adjuvant subcutaneous immunotherapy by local deposition of the allergen, preventing rapid entry to the systemic circulation (46). Another adjuvant used in AIT regimens is Microcrystalline Tyrosine (MCT). This is in part due to the biodegradable nature of MCT, while still maintaining depot formation, a crucial part of maintaining a sustained immune response during AIT. Reports of the efficacy of MCT have found it comparable with Alum (47, 48). In the currently available allergy vaccine, Pollinex Quattro MCT is the adjuvant that is mixed with a grass pollen allergoid and with the TLR4 agonist Monophosphoryl-lipid A (MPL) (discussed below) (49).

## Extracellular TLRs

### TLR2 Agonists

Human TLR2 is expressed as a heterodimer on the surface of plasma membranes, with two observable configurations; TLR2/6 and TLR2/1. The discovery of ligand specificity was made through studies in mouse models in which either TLR6 or TLR1 were knocked out and their responses to TLR ligands observed (50, 51). The association between TLR2 and asthma and allergy prevalence remains controversial, with recent meta-analyses revealing that the TLR2 polymorphism rs3804099 can be indicative of asthma risk or has no association (52, 53). TLR1 and TLR6 polymorphisms, however, have been reported to be associated with asthma and atopy in early life (54). At the protein level, TLR2 expression is upregulated in the nasal mucosa of patients with persistent AR and maybe an aggravating factor in the disease (55).

### TLR2/1

The immune potentiating activity of synthetic TLR2 agonist Pam3Cys was first described through the induction of IL-1 from human mononuclear cells (56), subsequently being highlighted as a potential immunoadjuvant in the Pam3Cys-Ser-(Lys)4 (Pam3CSK4) analogue (57). Pam3Cys is specific to TLR2/1, with TLR6 deficient mice demonstrating an ability to produce a normal response to the ligand, whereas TLR2 deficient and TLR1 deficient mice either did not respond or produced a compromised response respectively (50, 51).

Since their characterisation, the Pam3Cys analogues have been used in numerous and conflicting studies with evidence put forward of the capacity to induce polarization towards a Th1 or Th2 phenotype in both mice and humans. Characterisation of the nature of the immune response induced by Pam3Cys-Ala-Gly in human DCs first revealed that the peptide favours induction of a Th2 phenotype through the production of IL-12p40 (58) and upregulated IL-5 and IL-13 production from naïve CD4+ cells that were co-cultured with Pam3Cys pre-treated DCs (59). Contrary to these findings, Pam3CSK4 alone did not induce IL-5 and IL-13 from human PBMCs and in fact, suppressed house dust mite induced IL-5 and IL-13 (60). The lack of IFN-γ production suggests that this reduction may not be Th1 mediated, however, and there may be a different underlying mechanism. In a model of birch pollen allergy, CD4+ naïve cells were observed to shift towards both Th1 and Th2 phenotypes, with upregulation of both IL-4 and IL-13 (61). It was also observed in this study that Pam3CSK4 enhanced allergen uptake to CD14+ cells and DC maturation compared to allergen alone, a finding that had also been observed in neonatal cord blood cells (62). The effects of TLR2/1 agonists in humans have not been investigated and *in vivo* effects of the capacity of these agonists to modulate tolerance have only been observed in mice.

In murine studies of ovalbumin (OVA) induced experimental asthma, co-administration of Pam3Cys revealed that Pam3Cys potentiated the Th2 response rather than preventing or reducing it (63), although this is the only study that observed preferential induction of a Th2 response. A subsequent study demonstrated that Pam3CSK4 reversed OVA-induced allergic inflammation and promoted a deviation towards a Th1 response through upregulation of IL-12 rather than through induction of Tregs (64). A later study however, highlighted the role of Tregs in addition to Th1 deviation (65). This role for Tregs was further demonstrated in a murine OVA model where Pam3Cys resulted in the expansion of Tregs in parallel with a decrease in eosinophilic infiltration in the airways, and a reduction in airway hyperresponsiveness to methacholine (66). An alternative mechanism of TLR2/1 downregulation of Th2 responses was suggested in a study that demonstrated that Pam3CSK4 induced tolerance through apoptosis of CD4+ T cells, as observed in splenocytes (67, 68).

The conflicting nature of the information generated regarding the capacity of TLR2/1 agonists to attenuate allergic inflammation is a consequence of the different agonists used and the models they are tested in. Collectively however, human *in vitro* studies and murine *in vivo* studies support the potential for TLR2/1 agonists as adjuvants for immunotherapy, with observed consistent downregulation of allergen-induced Th2 responses although the underlying mechanism remains controversial.

### TLR2/6

Macrophage activating lipopeptide of 2 kDa (MALP-2) was first identified in 1997 as a naturally occurring lipopeptide derived from mycoplasma bacteria (69), although subsequent studies investigating its actions have used a synthetic version. As a dicacylated lipopeptide, it targets the TLR2/6 receptor, as confirmed in a TLR6 knockout model (50). In a mouse model, co-administration with the antigen beta-galactosidase (beta-gal), MALP-2 resulted in a dominant Th2 response that could limit its suitability as an adjuvant for allergen immunotherapy (70).

The first study in human cells highlighted the potential of MALP-2 to stimulate DCs leading to a downstream proliferation of lymphocytes and the production of cytokines, including IL-4, IL-5 and IFN-γ (71). A subsequent study further expanded on this by showing that DCs from house dust mite allergic patients primed with IFN-γ and MALP-2 would provide a shift of Th2 lymphocytes towards Th1 as determined by the ratio of IFN-γ and IL-4 production (72). In the absence of IFN-γ, however, the lymphocyte response favoured Th2, which suggested that the co-administration of MALP-2 as an adjuvant with allergen would be insufficient to skew the allergic response. MALP-2 has also been investigated as the synthetic bisacycloxypropylcysteine derivative conjugated with polyethylene glycol (BPPcysMPEG) that demonstrated improved solubility and stability over MALP-2 alone. In a human *in vitro* model utilising cells from patients with a history of grass pollen allergy, BBPCysMPEG actually enhanced allergen-induced IL-5 production (73).

In animal models, MALP-2 was shown to downregulate Th2 responses in OVA-induced airway hyperresponsiveness when animals were treated with MALP-2 and IFN-γ after sensitisation (74) although the effectiveness of MALP-2 administration in the absence of IFN-γ again was lacking. When a MALP-2 derivative was co-administered in a mouse model of timothy grass pollen-induced allergy, a reduction of eosinophil infiltration was observed, in conjunction with a reduction in IL-4 and IL-5 in BALF (75). This was observed despite the lack of a pronounced IL-10 mediated Treg or IFN-γ mediated Th1 response to the agonist. In *in vitro* studies of the effects of MALP-2 on peritoneal mast cells from mice and increased in IL-10 secretion over pro-inflammatory cytokines combined with an up-regulation of IFN-γ was observed (76). These data suggested that the omission of granulocytes in different studies may account for lack of IFN-γ and IL-10 activity. In an *in vivo* sensitisation model with Phlp p 5 allergen, BPPCysMPEG immunotherapy reduced infiltration of lymphocytes and eosinophils into the lungs, with similar reductions in IL-4 and IL-5 levels (73). A subsequent study using OVA also demonstrated a reduction in airway inflammation through diminished production of IL-5 mediated by enhanced IFN-γ secretion (77).

As with TLR2/1, TLR2/6 agonists do show a down-regulation of Th2 cytokines, and this seems to be IFN-γ mediated. Despite the early observation TLR2/6 required co-administration of IFN-γ to achieve efficacy, studies published later do not support this observation (73, 76, 77). Therefore, both TLR2/1 and TLR2/6 agonists may be viable for further investigation for use as adjuvants in immunotherapy.

**Figure 4:**
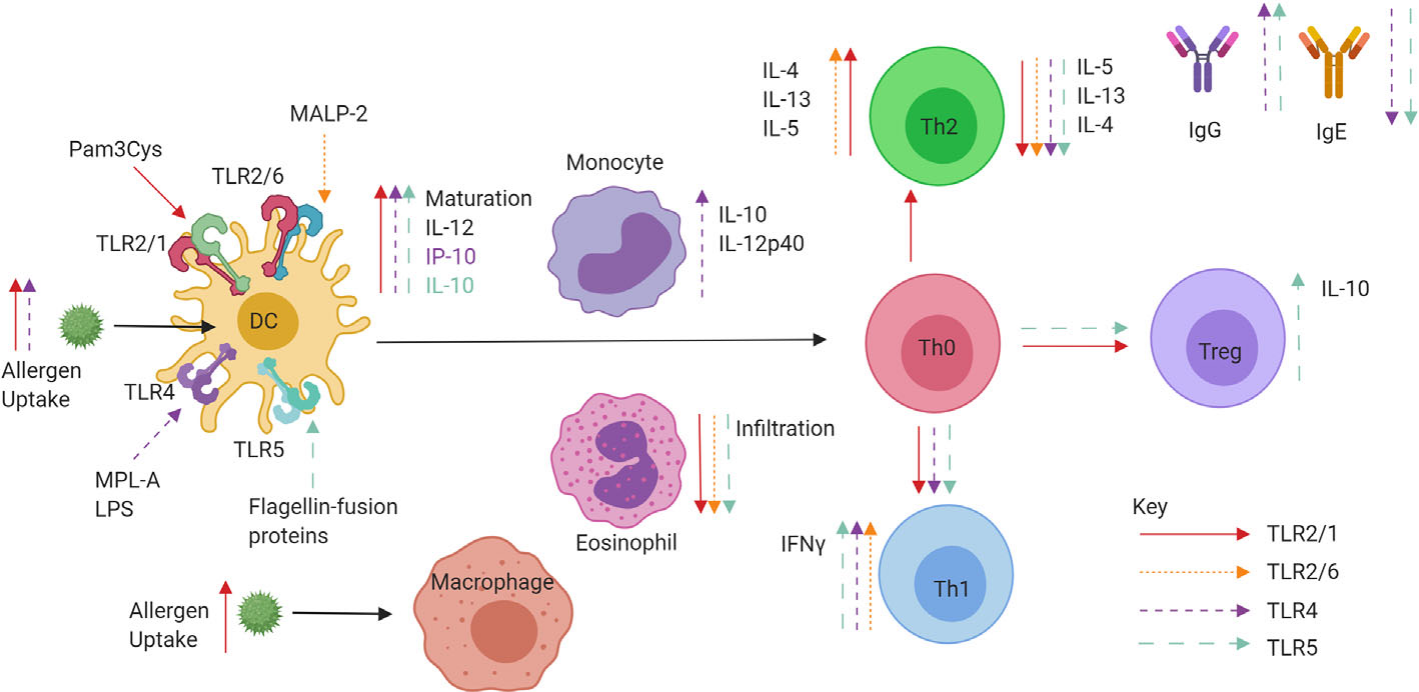
Role of extracellular TLR agonists when administered in conjunction with allergen or conjugated to allergens in a fusion protein. TLR2/1 (red), TLR2/6 (pink), TLR4 (purple), and TLR5 (blue) specific effects of tolerance induction. Ligand binding to TLRs on DCs can promote maturation, IL-10 and IL-12 production leading to priming of Naïve CD4^+^ T helper cells. The promotion of IFNγ producing Th1 cells and IL-10 producing Tregs is observed, as well as increases in IgG and decreases in IgE. Both decreases and increases in Th2 cell cytokine production are also observed.

TLR2 research into respiratory diseases over the past two decades has not progressed beyond *in vitro* human or *in vivo* mouse models. While some studies report enhancement of a Th2 allergic response, the majority favour a reduction of Th2 cytokines, cellular infiltrates in BAL fluid and upregulation of Th1 cytokine production and Treg proliferation. Future work and direction would ideally see the development of a suitable candidate for administration in humans, and it is possible that TLR2 agonists currently used therapeutically such as OK-432 for cancer, may be viable treatments for AR and asthma (78). A summary of TLR2/6 and TLR2/1 effects on allergic inflammation can be found in **Figure 4**.

### TLR4

The first identified TLR, TLR4 is expressed on plasma membranes and responds to stimulation with bacterial lipopolysaccharide (LPS). A substantially less toxic derivative of LPS is the agonist MPL, which has been used in numerous adjuvant systems for a wide range of diseases ranging from cancer to infection. MPL is a component of the adjuvant Pollinex Quattro, which has undergone randomised clinical trials in allergic rhinitis in man. However, there is not a clear indication as to the role of TLR4 in asthma nor atopic diseases, with meta-analyses having identified that TLR4 polymorphisms may be either preventative (79) or represent risk factors (53) for development of asthma while TLR4 protein levels are enhanced in the nasal mucosa of patients with allergic rhinitis (55). Whilst MPL has been used clinically as an adjuvant, lipopolysaccharide (LPS) has been used in the majority of *in vitro* and animal mechanistic studies.

Characterisation of the role of LPS in human DCs revealed that LPS promotes the production of IL-12p70 and IP-10, providing a potent Th1-biased stimulus (58). IL-12p70 was then demonstrated to provide a favourable ratio of IFN-γ to IL-5 and IL-13 in pre-treated human DCs co-cultured with naïve CD4+ cells (59). In an *in vitro* model of birch pollen allergy that involved birch pollen stimulation of human cord blood cells, downregulation of Th2 responses was observed with a reduction in IL-13 following LPS co-administration (62). The timing of LPS administration in relation to allergen appeared to be a crucial factor. For example, following seasonal allergen exposure, an increase in TLR4 expression in nasal lavage cells was observed. When the nasal challenge was performed with allergen followed by LPS, an increase in both Th1 and Th2 cytokines in the nasal fluid was observed, whereas this was not observed when LPS preceded the allergen challenge (80). Studies using human PBMCs stimulated with the less toxic TLR4 agonist MPL demonstrated increased secretion of IL-10 and IL-12 (81). A subsequent study that combined TLR4 agonist MPL with grass pollen allergen *in vitro* resulted in downregulation of IL-5 and up-regulation of IFN-y production (82). This inhibition of Th2 cytokine release by PBMCs was shown to be both IL-12 dependent and dependent on the presence of monocytes as antigen presenting cells (82). A subsequent study with human monocytes and monocyte-derived DCs observed an enhancement of allergen uptake, DC maturation and the promotion of IFN-y producing Th1 cells, although there was no induction of IL-10 from T cells (61).

In a murine model, the administration of MPL during simultaneous sensitisation to OVA or ragweed pollen upregulated IgG antibodies and induced a Th1 response while in the absence of MPL only IgE was increased (83). In a separate model involving co-administration of LPS with the allergen, the use of an IL-12 knockout showed that the mechanism of LPS polarisation towards a Th1 immune response was IL-12 independent (84). An alternative study with LPS found that the skewing of Th2/Th1 balance in favour of Th1 was found to be IFN-γ-mediated (68). LPS has also been shown to promote suppression of Th2 mediated allergic responses through NO synthase two activity, suggesting the anti-allergic effects of LPS are not entirely mediated *via* Th1 and IFN-γ (85). An important consideration is the concentration of agonists administered, as low doses of LPS have been observed to promote Th2 responses during allergen sensitisation, whereas higher doses promoted preferential Th1 responses to the allergen (86). A recent study utilised the synthetic TLR4 agonist glucopyranosyl lipid A (GLA) as an adjuvant in a mouse model of Japanese Cedar Pollen induced AR. Utilising GLA, a suppression of eosinophilia, sneezing and IL-5 secretion were observed although the mechanism was not explored (87). Pollinex Quattro is a glutaraldehyde-modified grass pollen allergoid that is co-administered with MPL along with microcrystalline tyrosine (49). The combination has been shown in clinical trials to induce IgG production, whilst simultaneously reducing allergen-specific IgE levels in serum (88–90). Other studies in children with seasonal rhinitis and asthma, whilst confirming increases in allergen-specific IgG4, demonstrated no parallel reductions in allergen-specific IgE (91). A re-formulation of Pollinex Quattro, MATA-MPL (Modified Allergen Tyrosine Adsorbate—MPL) has been used in clinical trials of both grass and ragweed allergy. In a randomised double-blind placebo-controlled trial (RDBPCT), the grass pollen MATA-MPL-treated group demonstrated significantly reduced symptoms during the pollen season, accompanied by increases in allergen-specific IgG (92). In the absence of a conventional immunotherapy control group, no conclusions could be drawn regarding relative efficacy, although an advantage was the reduced number of injections compared to conventional regimens. In a separate RDBPCT for ragweed allergy, a 13% reduction in seasonal symptom scores compared to placebo was accompanied by increases in allergen-specific IgG, IgG1 and IgG4 (93). In a small phase I/IIa RDBPCT, a sublingual combination of allergen and MPL showed a trend for a reduction in the proportion of positive nasal challenge tests and reduced IgE and enhanced IgG compared to grass pollen sublingual immunotherapy alone (94). A recent study of MPL targeted patients that had received a course of grass pollen AIT completed five years before enrolment. Patients were administered either symptomatic medication or AIT consisting of tyrosine-absorbed grass pollen allergoid with MPL. A significant decrease in combined symptom medication scores for the AIT-MPL group was observed over the control group (95) suggesting potential for the use of MPL-adjuvanted allergoid as a booster for use following AIT withdrawal. A further small-scale *in vitro* study in birch pollen allergic patients has identified MPL as potentiating allergoid responses in subcutaneous immunotherapy (96). Further studies are required to explore the long-term effects of MPL-adjuvanted allergoid and also direct comparative studies with conventional vaccines. A summary of TLR4 effects on allergic inflammation can be found in **Figure 4**.

### TLR5

Mammalian TLR5 is expressed within the plasma membranes of many cells types both within and outside of the immune system and plays a key role in maintaining intestinal homeostasis. Identified by its capacity to recognise flagellin, expressed on both gram-negative and gram-positive bacteria, TLR5 provides the host with the capacity to detect a broad range of pathogenic bacteria (97). One study investigating a potential role for TLR5 in allergy used flagellin to stimulate cord blood mononuclear cells. Cells from new-borns that would later develop IgE-mediated allergic disease produced significantly higher TNF-α and IL-6 to those that would not (98). There currently remains no evidence, however, that TLR5 polymorphisms may influence allergy or asthma risk. Recent studies of TLR5 activation as a strategy for treating allergic disease have used a TLR5 fusion protein conjugated with different allergens to explore the impact on T cell phenotype and downregulation of the Th2 response and the durability of the effect.

Unlike TLR2 and TLR4 agonists, *in vitro* studies showed that flagellin did not enhance allergen uptake in monocytes and mDCs from allergic donors (61). However, when cultured with naïve CD4+ cells, enhanced secretion of IFN-γ, IL-5, and IL-13 was observed through the generation of Th1 and Th0 cells. Subsequent studies have since analysed fusion proteins of flagellin and allergens to assess their capacity to alter human allergic responses. In PBMCs from patients with house dust mite induced asthma, an OVA/Flagellin fusion protein inhibited an inflammatory response through IL-10 production from regulatory DCs that promoted Treg enhancement while diminishing Th1/Th2 responses (99). Another study using *in vitro* human PBMCs from allergic patients assessed Bet v one allergen genetically fused to flagellin. The fusion protein enhanced the capacity of DCs to stimulate T cell responses towards IFN-γ production and reduced IgE binding, demonstrating reduced allergenicity and enhanced immunogenicity (100). The fusion protein rFlaA: Betv1 was used to stimulate PBMCs from birch-allergic subjects. The rFlaA: Betv1 protein activated the known mammalian target of rapamycin in mDCs to promote IL-10 secretion that inhibited the production of Th2 cytokines such as IL-13 (101).

In mice, flagellin alone was observed; paradoxically; to enhance allergen-induced Th2 responses (102). Another study confirmed that flagellin co-administration alone rather than as a fusion protein-induced Th2 responses through induction of IL-33 and TSLP secretion from lung epithelial cells (103). Therefore, it is likely that any benefit to be gained from utilising flagellin in AIT, would be through its use as a fusion protein.

Fusion protein studies commenced earlier in mice than *in vitro* human investigations, the first of which saw mice sensitised to OVA, followed by treatment with OVA-FlaB and then challenged. The treatment reduced eosinophil infiltrate, airway hyperresponsiveness, IL-5 and IL-4 production from splenocytes (104). This suppressive capacity was also observed through the suppression of IgE and IgGa2, mediated through enhanced levels of *in vivo* IL-10 (105). Epithelial cytokines IL-25 and IL-33, which are typically secreted upon allergen exposure, were also reduced following OVA-FlaB treatment (106). When flagellin was fused with birch pollen allergen Bet v 1 to create rFlaA: Betv1, a suppression IgE, production and Th2 responses were observed (101) The reduction of allergic responses and promotion of tolerance was demonstrated to be IL-10-dependent, and the cell source of IL-10 was identified as monocyte-derived DCs (107). This was confirmed when a different fusion protein of flagellin and allergen was shown to inhibit OVA-induced allergic asthma through the activity of Tregs and regulatory DCs (99). Finally, mugwort allergen art v 1 fused with flagellin to make fusion protein rFlaA: Betv1 induced mammalian target of rapamycin dependent IL-10 secretion from DCs and enhanced production of IgG1 and IgG2a detected from serum and IFN-γ (108).

While Flagellin has been identified as an aggravating factor in allergic airway disease, it is clear that utilisation of the protein fused to an allergen has a potent capacity to reduce allergic responses and could potentially be developed as an adjuvant for AIT. A summary of TLR5 effects on allergic inflammation can be found in **Figure 4**.

## Intracellular TLRs

### TLR3

TLR3 was identified as the first TLR capable of binding viral components through stimulation of the receptor with Polyinosinic:polycytidylic acid (poly I:C), a synthetic form of double-stranded RNA (dsRNA) (109). TLR3 was subsequently discovered to reside within the intracellular endosomal compartment (110). There is limited information available that might link with susceptibility to allergy. However, polymorphisms have been identified to associate with specific asthma phenotypes, but these do not appear to impact on overall asthma susceptibility or severity (111).

In human bronchial epithelial cells from healthy donors, stimulation of TLR3 has been shown to promote the secretion of TSLP (112) a key cytokine involved in priming DCs to promote Th2 immune responses. Capsazenoids are proteins that stimulate Transient receptor cation family subfamily V member 1 (TRPV1) nociceptive receptors present in human bronchial epithelium. Capsazenoids have also been shown to inhibit TLR3-mediated production of TSLP from human bronchial epithelial cells (113, 114). However, it remains unclear if this would be a therapeutically viable alternative as an adjuvant to AIT.

**Figure 5:**
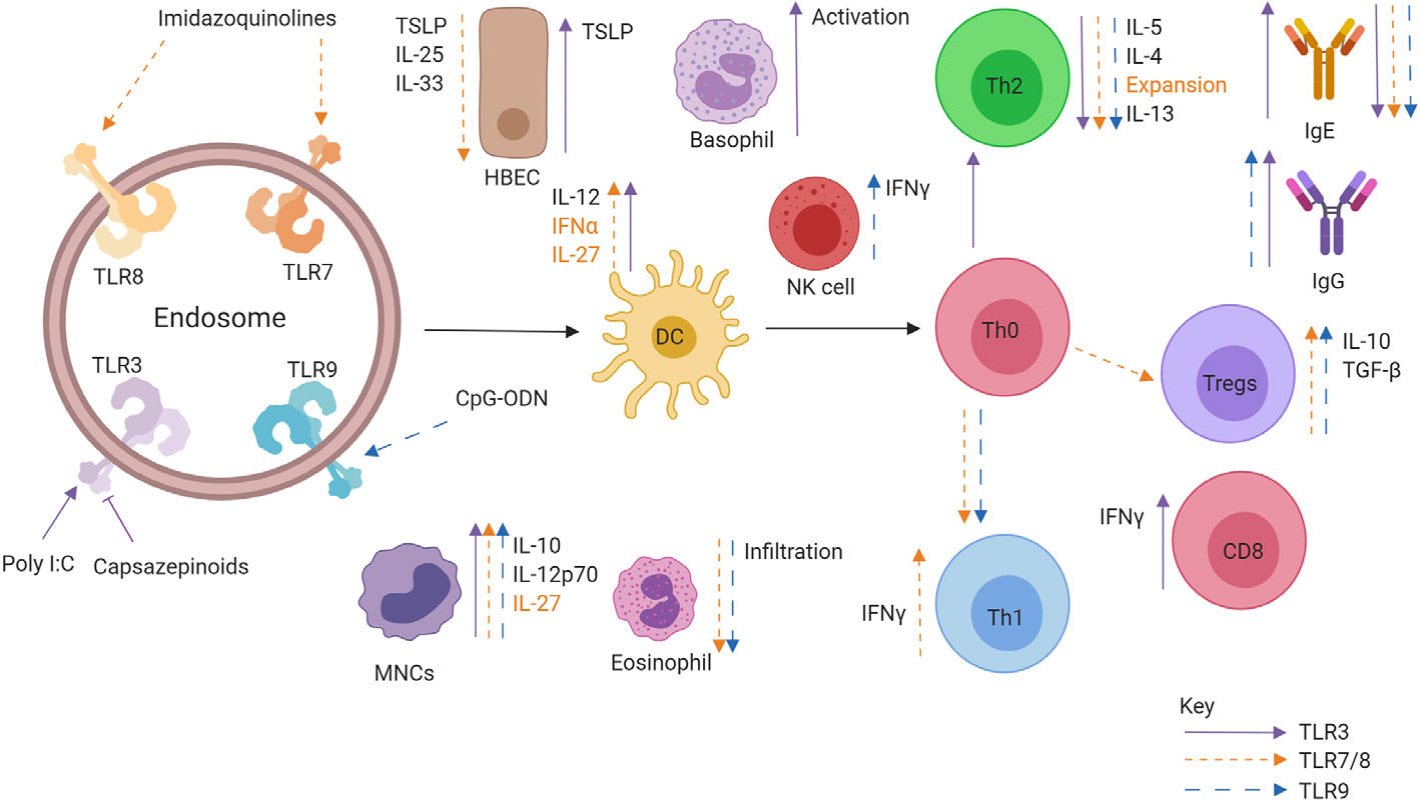
Role of Intracellular TLR agonists when administered in conjunction allergen. Activation of TLR7/8 (orange), TLR 3 (purple), and TLR9 (blue) leads to specific effects promoting downstream allergen tolerance. TLR activation can promote IFNα, IL-12, and IL-27 production from DCs, leading to the generation of a Th1 and Treg response producing IFNγ and IL-10, respectively. There is a marked decrease in Th2 cytokine production as well as alarmin secretion from epithelial cells. Allergen specific IgE is also downregulated while allergen specific IgG is upregulated.

Studies in mice investigating stimulation of TLR3 in combination with allergen sensitisation have generated an unclear picture on the role of TLR3, with studies advocating both reductions and enhancement of allergic symptoms. Co-administration of Poly I:C during sensitisation abolished the production of IL-5 and IgE, while administration after the sensitisation phase saw reductions in symptoms following subsequent exposure mediated through IL-12p70 and IL-10 (115). Polyadenylic:polyuridylic acid (poly A:U), a form of Poly I:C with reduced toxicity, induced secretion of IL-12p40 from DCs and IFN-γ from CD8+ T cells in mice (116). However, it remains unclear if CD8+ T cells may promote or attenuate allergic airway inflammation (117). The production of IL12-p40 suggests this may favour Th2 allergic inflammation due to the competition with the active heterodimer IL-12p70 that promotes Th1 responses. Additional studies have demonstrated that TLR3 agonists promote or have no effect on allergic inflammation, with Poly I:C unable to reduce Th2 allergic responses in mice in a subcutaneous OVA sensitisation model with subsequent intranasal OVA allergen challenge (68). A previous study had also highlighted that Poly I:C was capable of enhancing allergic sensitisation through IgE and IgG1 production, suggesting TLR3 may promote rather than attenuate allergic airway disease (118). In an *in vivo* sensitisation model, mice were administered OVA *via* the subcutaneous route prior to intranasal challenge with OVA followed by Poly A: U after one hour. In this setting, Poly A: U exacerbated OVA-induced allergic asthma through basophil activation *in vitro* and *in vivo* (119). A summary of TLR3 effects on allergic inflammation can be found in **Figure 5**.

The most promising therapeutically relevant data was obtained when TLR3 was inhibited *via* the use of Capsazenoids (113, 114). However, as there are limited studies upon the actions of TLR3 activation or inhibition in asthma and allergy, a conclusion cannot be drawn about either strategies inclusion in AIT. Coupled with no apparent association of TLR3 to the development or severity of either asthma or allergy, it is unlikely to be developed further in the future.

### TLR7/TLR8 Agonists

TLR7 is an intracellular TLR located with the endosomal compartment of immune cells. The discovery of TLR7 was made utilising the antiviral imidazoquinoline compound imiquimod (120). A later study then identified that single-stranded RNA was capable of activating both TLR7 and TLR8 (32). The key downstream effect of TLR7 activation is the production of type 1 interferons such as IFN-α, an essential cytokine in antiviral immunity and prevention of the development of Th2 responses (121). Both TLR7/TLR8 polymorphisms have been associated with atopy and asthma (122–124), and Type 1 interferons have also been implicated (125) making TLR7/8 agonists attractive compounds as adjuvants for AIT.

Imidazoquinoline compounds Imiquimod and Resiquimod (R-848) are tricyclic organic compounds with small molecular weights. The compounds are used in a variety of antiviral medications, while they have only been investigated *in vitro* or in animal models with regards to AR and asthma. The compounds have consistently demonstrated a capacity to reverse Th2 responses in favour of an antiallergic Th1 response. When administered to human PBMCs stimulated with the mitogen PHA, TLR7 agonists downregulated IL-4 and IL-5 whilst increasing IL-12 and IFN-γ (126). In an *in vitro* model of human DCs stimulated with TSLP, the conventional Th2-mediated response was ablated by the administration of Resiquimod. A promotion of IL-12 and IFN-γ contributed to Th1 immune deviation and IL-10 production (127). This phenotype switching has also been associated with a reduction in IL-4 as a consequence of IFN-α secretion, enabling polarisation towards a Th1 phenotype (128). Finally, when Resiquimod was added to human PBMCs stimulated with CD40-ligand and IL-4, there was an observed reduction in IgE synthesis that appeared to be due to a parallel induction of IFN-γ (129).

In both OVA-induced asthma models and allergy models in mice, the protective effect of the compounds has also been demonstrated. TLR7 activation enhanced levels of IFN-γ and IL-12 while reducing BAL infiltrate and inflammation (130), with these effects mediated through IL-12 and IL-10 (115). IL-27 production from DCs and monocytes by Resiquimod has also been observed and shown to be necessary to suppress Th2 allergic responses (131). Additionally, TLR7 activation promoted Treg expansion and secretion of IL-10 and TGF-β, with the removal of Tregs diminishing the suppressive effect of Resiquimod. Finally, Resiquimod also affected humoral immunity through inhibition of IgE synthesis from B cells (132). The suppressive effect of Resiquimod has also been suggested to be mediated through alternative factors to Tregs and Th1 upregulation. CD8+ and NKT cells have been highlighted as sources for IFN-γ production (133, 134). Resiquimod was demonstrated to reduce epithelial factors IL-25, IL-33, and IL-13 from the serum of OVA sensitised mice while promoting Th1 and Treg cell populations (135). Increased IgG2a has been observed as a protective mechanism, alongside reduced IgE, reduced IL-5, IL-4, and IL-13 (136). Another potential mechanism of TLR7 mediated suppression of allergic responses is a reduction of reactive oxygen species (ROS) through induction of antioxidant factors (137) accompanied by reduced contractile responses of airways when stimulated with potassium (138). In a contractile airway model in guinea pigs, TLR7 agonists decreased contractility indirectly through disruption of calcium homeostasis in the airway smooth muscle (139). One study utilised a nanoparticle formulation of an OVA peptide in the presence/absence of Resiquimod. The addition of Resiquimod resulted in suppression of *in vivo* allergic responses following OVA-food challenge. The mechanism likely involved the generation of TGF-β producing tolerogenic DCs that in turn, induced FoxP3+ expressing Tregs capable of suppressing the response to food challenge (140). It is not clear to what extent these responses observed in a murine oral challenge model may have applications in human respiratory allergy.

While TLR7 agonists are utilised in clinical settings outside allergy there a few studies highlighting their effects in modifying human allergic responses. GSK2245035, an adjuvant designed to be administered intranasally, has undergone structural activity relationship design to induce preferentially IFN-α over inflammatory factors such as TNF-α. In vitro studies with human PBMCs have demonstrated suppression of IL-5 cytokine responses to allergens while promoting IFN-γ and IL-10 production from human PBMCs (141). A RDBPCT demonstrated that the compound was well tolerated at 20 ng intranasal doses despite some incidences of flu-like symptoms developing (142). Reductions in nasal symptom scores after ragweed pollen nasal allergen challenge were observed at three weeks from baseline but were lost at one year (143). A novel approach to circumvent potential systemic toxicity issues with TLR7 administration has been the development of TLR7 “ante-drugs” which are metabolised upon entry into the systemic circulation to minimise toxicity and potential side effects. Administration of AZ12441970 to human PBMCs promoted IFN-α production and reduced IL-5 secretion (144). AZ12441970 reduced house dust mite induced IL-5 secretion from PBMCs through the actions of IFN-α on T cells, as well as through the upregulation of notch ligands (145). These ante-drugs were also observed to suppress allergic airway eosinophilia in mice through type 1 IFN secretion and not the promotion of type 2 interferon responses (146). Recently, the compound entered a small RDBPCT in which participants were administered four weekly inhaled doses *via* a nebuliser. Systemic interferon production and induction of flu-like symptoms were observed, potentially limiting the utility of the compound (147). A further trial has shown that while drug-related adverse events were frequent, they were mild and well-tolerated (148). The TLR7 agonist AZD8848 was shown to suppress the allergen-induced late-phase asthmatic response in an RDBPCT where participants were administered AZD8848 intranasally once-weekly for eight weeks. This suggests that the drug may have a prolonged immunomodulatory effect, although this was lost at four weeks. The effects of TLR7/8 agonists on allergic inflammation are illustrated in **Figure 5**.

### TLR9

TLR9 was recognised as being the receptor responsible for detecting and propagating immune responses to bacterial DNA, which contains high levels of CpG dinucelotides in contrast to mammalian DNA (149). A range of CpG-Oligodeoxynucleotides (CpG-ODNs) have been used as a tool for exploring the biology of the receptor, and several TLR9 agonists have been developed in combination with an allergen in clinical trials of adjuvanted immunotherapy. Common polymorphisms have been shown to have no association with the development of allergy (150) or asthma (151). However, the agonists have a strong capacity to induce Th1 mediated responses and consequently may provide benefit when administered as an adjuvant to AIT.

In a study contrasting the effects of CpG-ODN in the PBMCs of allergic and non-allergic individuals, the nature of CpG-ODN to induce Th1 response was observed to be unaffected by the atopic status of participants. An increase in IL-12 dependent IFN-γ secretion was observed with the source of IFN-γ identified as NK cells (152). In parallel, it was observed that allergen-induced IgE synthesis was reduced, while IgG and IgM production were enhanced. Another study utilised PBMCs from patients with AR, and it was observed that enhanced IFN-γ production was accompanied by an increase in IL-10 and reduced IL-4 production (153). It was also observed that when patients resumed corticosteroid and antihistamine treatment after initial sampling, the antiallergic response of PBMCs to CpG was enhanced (153). CpG-ODN was also shown to alter the allergic status of pDCs from allergic patients. pDCs primed with CpG ODN were co-cultured with CD4+ cells and promoted IFN-α and IFN-γ secretion. Without CpG-ODN, pDCs in co-culture preferentially promoted CD4+ helper Th2 responses (154). In a study that utilised cells from patients with Japanese Cedar Pollen allergy, B cells were treated with CpG-ODN before exposure to allergen stimulated CD4+ cells. An observed induction of PD-L1 on B cells was caused by CpG-ODN treatment, and this was accompanied by a reduction in IL-5 and IL-13 production while promoting IL-12 and IFN-γ production (155). This was observed to be dependent on PD-L1 binding to PD-1 as antibodies directed against PD-1 reversed the effect.

Similarly, in *in vivo* studies in mice, TLR9 agonists also stimulated Th1 mediated reductions in Th2 responses to allergens. Administration of CpG-ODN during sensitisation to OVA prevented the development of Th2 disease through reduction of IL-4, IL-5, IL-13, IgE eosinophilic inflammation and promoted IFN-γ production versus OVA alone (156). Similarly, when CpG-ODN was co-administered during sensitisation in a cockroach induced-allergic asthma model, eosinophilic inflammation, and airway hyperresponsiveness after subsequent allergen challenge were reduced. A parallel reduction in IL-5, IL-13, and IgE was accompanied by increases in IL-10 secreting Tregs and upregulation of Th1 responses (157). Similarly, CpG ODN1826 reduced airway eosinophilia, IL-4 and IL-5 levels, and increased IL-10 when administered during allergen sensitisation (158). When administered post-allergen sensitisation *via* the intranasal route, promising results were also observed with reduced eosinophil infiltration, IL-4 and IL-5 levels, serum levels of OVA-specific IgE and symptom scores (158). When administered *via* the intradermal route, this was not observed (159). As highlighted previously in humans, anti-allergic effects of CpG ODN were mediated through IL-12 promoted IFN-γ production (103). A recent murine study utilising the administration of CPG during sensitisation to OVA observed a decrease in IgE and promotion of IgG through a MyD88 dependent mechanism through cDCs that was independent of type 1 IFNs (160). This suppression of IgE generation through CpG was also observed in a study utilising the aeroallergen Blomia tropicalis and highlighted a potentially important observation that IL-10 is essential in preventing a full potentially harmful Th1 response following CpG treatment (161). CpG has also been shown to act on ILC2s, through a direct suppression mediated by IFN-γ that limits IL-33 production and consequently attenuates airway hyperresponsiveness (162). Animal studies using the ISS ligand observed reduced airway remodelling and cellular infiltration in sensitised mice when administered pre-allergen challenge (163). When ISS-ODN was conjugated with Der f dust mite a suppression of allergen-induced eosinophilia, IL-5 was observed along with an increase in IFN-γ and TGF-β (164).

Such has been the potency to induce Th1 responses, CpG based agonists have been investigated in early-stage clinical trials. In an RDBPC phase II trial of ragweed pollen conjugated to a phosphorothioate oligodeoxyribonucleotide immunostimulatory sequence of DNA (AIC), long term clinical efficacy was observed over two subsequent consecutive pollen seasons (165). Following six pre-seasonal injections, patients in the treatment group had lower symptom scores and improvements in quality of life during the pollen season compared to placebo. This was accompanied by a reduction in ragweed specific IgE and IL-4+ basophils. In a different study, nasal administration of CpG promoted Th1 mediated airway inflammation, although this was reduced in seasonal allergic rhinitis patients versus healthy controls (166). Subsequent trials have investigated the agonist QbG10, also referred to as CYT003. QbG10 administration, in conjunction with house dust mite during a Phase I/IIa clinical trial showed the agonist was well tolerated and induced tolerance to the allergen. Upon subsequent exposure to the allergen, patients remained symptom-free for 38 weeks, and allergen-specific IgG and IgE increased (167). Dual administration of CYT003-QbG10 in patients with allergic rhinoconjunctivitis in a phase IIb study showed an efficacious reduction in symptoms versus placebo (168). In asthma patients who received QbG10 injections without allergen while having steroid medication withdrawn, maintained well controlled-asthma versus those on placebo (169). Finally, CYT003 was used as a treatment for allergic asthma in an inhaled steroid during a phase 2b clinical trial and offered no significant improvement in symptom scores over placebo (170). The study was terminated following analysis of 12-week results.

There is no clear association with TLR9 expression or polymorphisms with asthma and atopy and it remains unclear if the agonist has potent clinical efficacy despite promising experimental findings. The use of the agonist during these early stage clinical trials also saw adverse events and so its use must be carefully considered. Further well-designed clinical trials of TLR9 agonists alone and in combination with allergen are required. A summary of TLR9 agonist effects on allergic inflammation can be found in **Figure 5**.

## Conclusions

Over the last two decades the discovery of novel, safe and effective forms of short-duration allergen immunotherapy for respiratory or food allergies has attracted considerable scientific interest. In this context, it has been suggested that the most promising solution may be the development of immunotherapy with adjuvanted-allergen formulations or combinations with immunomodulators that have the potential to suppress aberrant Th2 reactivity and promote adaptive immune responses capable of mediating a state of immunological tolerance to allergen exposure. To this end, several molecules with TLR-activating properties have been tested in pre-clinical models of allergic disease and few (i.e. TLR4 and TLR9 agonists) have reached the stage of clinical evaluation. Overall, the results from these studies indicate that this approach can safely boost the induction of protective immune mechanisms, including enhancement of Th1, Treg, blocking-antibodies, or other tolerogenic responses, with the particular up-regulated immune pathways and magnitude of benefit being TLR-ligand and route of administration specific. However, despite the demonstration of beneficial immunological changes, significant and persistent efficacy in clinical settings hast not yet been confirmed in large phase IIb or III clinical trials. The reasons for this curtailed success may have to do more with the shortened duration of the tested regimens, the level of the evaluated allergen doses or operational clinical trial issues and less with the immunomodulatory profile of the specific TLR agonists. Nevertheless, it is also possible that agents developed as adjuvants for vaccines against infectious disease (i.e. MPL) may not be the most appropriate potentiators of allergen immunotherapy. Further research is needed to optimise the adjuvanted-allergen immunotherapy regimens and to define the required immunological and pharmacological properties of molecules, including TLR ligands, which act as efficient enhancers of the desired outcomes of allergen immunotherapy, i.e. control of allergic reactivity/inflammation and induction of tolerance to allergens.

## Author Contributions

MK reviewed the literature, wrote the manuscript, and drew the figures. DT, SD, and MS contributed to the writing and correcting of the manuscript. All authors contributed to the article and approved the submitted version.

## Conflict of Interest

The authors declare that the research was conducted in the absence of any commercial or financial relationships that could be construed as a potential conflict of interest.
